# Intronic Alternative Splicing Regulators Identified by Comparative Genomics in Nematodes

**DOI:** 10.1371/journal.pcbi.0020086

**Published:** 2006-07-14

**Authors:** Jennifer L Kabat, Sergio Barberan-Soler, Paul McKenna, Hiram Clawson, Tracy Farrer, Alan M Zahler

**Affiliations:** 1 Department of Molecular, Cell, and Developmental Biology and Center for Molecular Biology of RNA, University of California Santa Cruz, Santa Cruz, California, United States of America; 2 Center for Biomolecular Science and Engineering, University of California Santa Cruz, Santa Cruz, California, United States of America; Washington University in St. Louis School of Medicine, United States of America

## Abstract

Many alternative splicing events are regulated by pentameric and hexameric intronic sequences that serve as binding sites for splicing regulatory factors. We hypothesized that intronic elements that regulate alternative splicing are under selective pressure for evolutionary conservation. Using a Wobble Aware Bulk Aligner genomic alignment of Caenorhabditis elegans and *Caenorhabditis briggsae,* we identified 147 alternatively spliced cassette exons that exhibit short regions of high nucleotide conservation in the introns flanking the alternative exon. In vivo experiments on the alternatively spliced *let-2* gene confirm that these conserved regions can be important for alternative splicing regulation. Conserved intronic element sequences were collected into a dataset and the occurrence of each pentamer and hexamer motif was counted. We compared the frequency of pentamers and hexamers in the conserved intronic elements to a dataset of all C. elegans intron sequences in order to identify short intronic motifs that are more likely to be associated with alternative splicing. High-scoring motifs were examined for upstream or downstream preferences in introns surrounding alternative exons. Many of the high- scoring nematode pentamer and hexamer motifs correspond to known mammalian splicing regulatory sequences, such as (T)GCATG, indicating that the mechanism of alternative splicing regulation is well conserved in metazoans. A comparison of the analysis of the conserved intronic elements, and analysis of the entire introns flanking these same exons, reveals that focusing on intronic conservation can increase the sensitivity of detecting putative splicing regulatory motifs. This approach also identified novel sequences whose role in splicing is under investigation and has allowed us to take a step forward in defining a catalog of splicing regulatory elements for an organism. In vivo experiments confirm that one novel high-scoring sequence from our analysis, (T)CTATC, is important for alternative splicing regulation of the *unc-52* gene.

## Synopsis

Alternative splicing of precursor messenger RNA is a process by which multiple protein isoforms are generated from a single gene. As many as 60% of human genes are processed in this manner, creating tissue-specific isoforms of proteins that may be a key factor in regulating the complexity of our physiology. One of the major challenges to understanding this process is to identify the sequences on the precursor messenger RNA responsible for splicing regulation. Some of these regulatory sequences occur in regions that are spliced out (called introns). This study tested the hypothesis that there should be evolutionary pressure to maintain these intronic regulatory sequences, even though intron sequence is non-coding and rapidly diverges between species. The authors employed a genomic alignment of two roundworms, Caenorhabditis elegans and *Caenorhabditis briggsae,* to investigate the regulation of alternative splicing. By examining evolutionarily conserved stretches of introns flanking alternatively spliced exons, the authors identified and functionally confirmed splicing regulatory sequences. Many of the top scoring sequences match known mammalian regulators, suggesting the alternative splicing regulatory mechanism is conserved across all metazoans. Other sequences were not previously identified in mammals and may represent new alternative splicing regulatory elements in higher organisms or ones that may be specific to worms.

## Introduction

One of the interesting lessons learned from the analysis of the human genome is that we may possess fewer than 25,000 genes [1]. One mechanism to dramatically increase the complexity of the human proteome from this lower-than-expected number of genes is to allow some genes to encode multiple proteins. This process can be accomplished by alternative precursor messenger RNA (pre-mRNA) splicing. Studies that use expressed sequence tag (EST) alignments to identify alternatively spliced genes have led researchers to predict that up to 60% of human genes are alternatively spliced [2–5]. Alternative splicing events can be regulated in tissue-specific, developmental, and hormone-responsive manners, providing additional mechanisms for the regulation of gene expression [6,7]. Understanding alternative splicing and its regulation is a key component to understanding metazoan genomes.

The current models for alternative splicing regulation are based on the interactions of intronic or exonic RNA sequences, known as *cis* elements, with splicing regulatory proteins known as *trans*-acting splicing factors [8]. The binding of splicing factors to the pre-mRNA regulates the ability of the spliceosomal machinery to recognize and promote alternative splicing. The role of intronic elements in regulating splicing is well established and has been shown to work in a combinatorial fashion based on the *trans*-acting factors that are present. For example, the inclusion of the 18-nucleotide-long, neural-specific N1 exon of the human *c-SRC* gene is regulated by the downstream control sequence found in the intron downstream of the N1 exon. This sequence serves as a recruitment site for both constitutive and neuronal cell-specific splicing factors such as nPTB, FOX-1, and FOX-2 [9–12]. The vertebrate RNA-binding protein FOX-1 can also regulate muscle-specific alternative splicing through interactions with the RNA sequence GCAUG [13], and repeats of this sequence have been shown to be important for alternative splicing regulation of the fibronectin exon EIIIB and the rat calcitonin/CGRP exon 4 [14,15]. Many other examples of complex and combinatorial regulation of alternative splicing through intronic *cis* elements have been demonstrated, and combinatorial interactions between proteins such as Nova-1, polypyrimidine tract binding protein (PTB), and ETR-3, with specific *cis* sequences, are important for alternative splicing regulation [16–20].

Intronic sequences are non-coding, and therefore they should have less evolutionary selective pressure to maintain their sequence. An exception to this should be intronic sequences that regulate alternative splicing. In an analysis of alternatively spliced human cassette exons, it was found that on average, approximately 100 nucleotides of intron sequence, flanking either side of the exon, tend to be highly conserved between the mouse and human genomes, with 88% identity in the upstream sequences and 80% identity in the downstream sequences [21]. Some clues to potential splicing regulatory motifs arise from these studies. For example, Sorek and Ast found that the sequence TGCATG was the second most common hexamer in the first 100 nucleotides downstream of alternatively spliced exons, appearing in 18% of these intronic regions [21]. Another study of aligned mouse/human alternative exons found that GCATG is the most overrepresented pentamer in the proximal conserved region of the intron downstream of alternative exons [22]. A third study found that TGCATG is frequently located in introns flanking brain-enriched alternative exons, and its presence and spacing are highly conserved in these genes from fish to man [23].

Using the nematode Caenorhabditis elegans as a model system, we have been working to take advantage of comparative genomics to identify *cis*-acting regulators of alternative splicing. The C. elegans gene structure, splicing machinery, and gene expression regulation is similar to that of other higher eukaryotes, with the exception that the average intron size is smaller. Our lab has previously developed methods for identification of alternatively spliced genes in C. elegans by aligning the genome sequence with ESTs and mRNA sequence [24]. We developed an algorithm, the Wobble Aware Bulk Aligner (WABA), for creating interspecies genome alignments between C. elegans and the related roundworm, Caenorhabditis briggsae [25]. WABA employs a hidden Markov model (HMM) to identify aligned regions as coding, high homology, low homology, and no homology. It also factors the AT richness of C. elegans introns into its calculations when it defines an intronic region as “high” homology [25]. C. briggsae and C. elegans diverged approximately 100 million years ago, yet are indistinguishable by eye [26]. Alignment of these two genomes revealed that exonic sequences are highly conserved between these species, but intronic and intergenic sequences are rarely conserved [25]. We found that these rare, high homology sequences in introns are more likely to occur in the introns flanking alternatively spliced exons than in total introns [25]. We hypothesized that these conserved intronic regions were *cis*-acting regulatory elements for alternative splicing. This nematode alignment, with relatively limited regions of high homology, provides the possibility for more specific pinpointing of intronic splicing regulatory elements than the much longer 100-nucleotide-long conserved regions flanking alternative exons in mouse/human alignments [21].

In this paper, we present the analysis of conserved regions of introns flanking alternatively spliced exons in C. elegans and correlate these conserved regions with alternative splicing regulation. We collected these conserved sequence regions into a database and searched for overrepresented pentamers and hexamers relative to a total intron database, similar to the method used by Burge's group to identify exonic splicing enhancers [27]. This allowed us to create a table of potential intronic alternative splicing *cis*-regulatory motifs. Since many RNA recognition motif–containing splicing factors recognize specific sequences on the order of 4–6 nucleotides in length [11,13,18,28–32], the high-scoring motifs in this catalog may represent specific binding sites for particular splicing factors. Several of our highest scoring motifs in this analysis correlate with known vertebrate splicing regulatory elements, for example, (T)GCATG [23], but several have not been previously identified. A number of candidates identified by this method were tested in an in vivo splicing reporter system in C. elegans. We have used this analysis to identify and confirm a new, highly conserved, alternative splicing regulatory element, (T)CTATC. We show that this sequence works in coordination with GCATG to regulate the alternative splicing of the *unc-52* gene.

## Results

### Identification of Highly Conserved Regions in Introns Flanking Alternatively Spliced Exons

In order to identify alternatively spliced cassette exons in the C. elegans genome, we used the Intronerator [24] to generate an initial set of 1,471 putative alternatively spliced genes. We did this by aligning over 200,000 ESTs and mRNAs to the C. elegans genome and identifying regions where the alignments are consistent with more than one way to process a gene. The program could not distinguish between alternative splicing and alternative promoters, so we analyzed each of these alignments individually to verify alternative splicing. We found 449 examples of genes with strong cDNA evidence for alternative cassette exons, and 454 examples of genes with alternative promoters, which usually had unique first exons that are spliced to common second exons. Of the genes with alternative splicing, 162 also contained alternative promoter usage. The remaining genes in the initial set of putative alternatively spliced genes were mostly due to unusual ESTs in the database that did not fit a gene model. We also saw evidence of ESTs that showed internal deletions at short direct repeats. To the program, these indicated the potential for intron removal, but upon further inspection, these did not meet the criteria of introns (start with the dinucleotides GT or GC and end with AG) and were likely the result of cloning artifacts of the ESTs.

**Figure 1:**
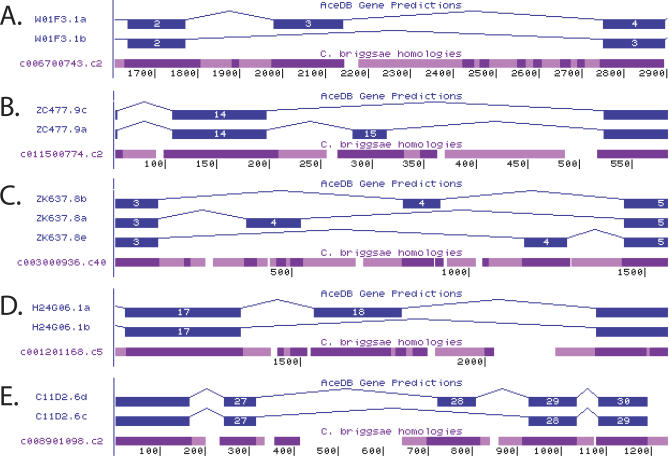
Images from the Intronerator Genome Browser Showing Alternatively Spliced Genes Gene isoforms predicted by the Wormbase Consortium are shown in blue, and WABA homology alignments for C. briggsae to this region of the C. elegans genome are shown in purple. Dark purple indicates a region of WABA high homology, light purple corresponds to low homology, and white indicates no homology between species. Regions of alternatively spliced genes: (A) W01F3.1, (B) ZC477.9, (C) ZK637.8, (D) H24G06.1, and (E) C11D2.6 are shown.

Intronic regions that are highly conserved between C. elegans and C. briggsae are rarely identified by WABA [25]. While analyzing our alternative cassette exon database, we were struck by the fact that we could identify many examples of WABA-defined high homology sequences in introns flanking the alternatively spliced cassette exons, suggesting strong evolutionary conservation. For 142 of the 449 alternatively spliced genes, WABA identified a highly conserved sequence in flanking introns upstream or downstream of 147 alternative cassette exons. Figure 1 shows several examples of screen shots from the Intronerator browser of cDNA-confirmed C. elegans alternatively spliced isoforms (in blue) and a graphical representation of the WABA alignments between C. elegans and C. briggsae (in purple). Dark purple regions, indicating high homology, can be seen in these introns either upstream or downstream of the alternative exon. Sometimes a single conserved element is identified by WABA, while for other cases multiple regions in the introns are conserved.

An important question is whether these 147 C. elegans alternative cassette exons are also alternatively spliced in *C. briggsae.* Due to a lack of C. briggsae transcript data, we have no direct evidence for alternative splicing of these exons. Therefore, we examined the C. briggsae homologs of these alternative exons for features of functional exons. A previous alignment of 8 MB of the C. briggsae genome with the complete C. elegans genome found that in coding exons there is 79.3% overall nucleotide identity [25]. Consistent with these findings, cross-species alignment of these 147 alternative cassette exons revealed an average nucleotide identity between C. elegans and C. briggsae of 81.8%. Amino acid identity of the open reading frame of these exons was 85.4%. Maintenance of the reading frame for these exons also appears to be well conserved: 57.0% of the 147 exons are the exact same size, 34.3% differ by a multiple of three nucleotides, and only 8.8% differ by a non-multiple of three nucleotides (unpublished data). The high conservation of nucleotide sequence, amino acid sequence, and maintenance of open reading frame is consistent with these being coding exons in C. briggsae.

One feature of alternative exons is that they are often flanked by weak splice sites which allow for splicing regulation (reviewed in [33]). If these exons are alternatively spliced in C. briggsae we would expect that they would be flanked by 3′ and 5′ splice sites of similar strength. We used the UCSC Genome Table Browser (http://www.genome.ucsc.edu) in an attempt to automate rapid alignment of the splice junctions for these 147 exons in C. elegans and C. briggsae. For the majority of these alternative exons, this automated method was successful in identifying C. elegans and C. briggsae 5′ and 3′ splice sites for each exon, and we used these data to compare the similarity of splice sites between the two species. For 5′ splice sites we examined the last three nucleotides of the exon and first six nucleotides of the intron, as these base-pair with U1 small nuclear RNA during initial 5′ splice-site recognition[31]. We found that these nine nucleotides were completely identical for 47.1% of the alternative exons and differed by only one nucleotide in 27.1% of the exons. For the 3′ splice sites, we examined the conservation of the last six nucleotides of the intron and the first nucleotide of the exon, as these are a binding site for the C. elegans homolog of the heterodimeric U2 auxiliary factor (U2AF) involved in initial 3′ splice-site recognition [34]. For 55.4% of the alternative exons examined, these seven nucleotides were completely identical, and for an additional 30.1% they differed by only one nucleotide. This strong conservation of splice-site sequence strength, along with regions of conservation in flanking introns, suggests that these exons may be substrates for alternative splicing in C. briggsae.

### The Role of Conserved Elements in Alternative Splicing Regulation

**Figure 2:**
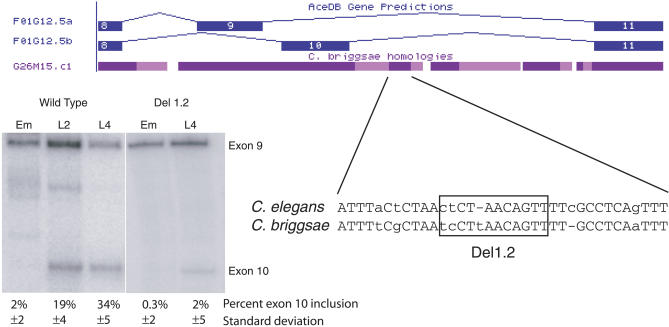
An Intronic Element Regulates *let-2* Alternative Splicing The top of this figure shows the alignment of C. elegans and C. briggsae sequences for the alternatively spliced region of *let-2*. Blue tracks indicate the splicing for the embryonic (top) and adult (bottom) isoforms. The purple track indicates homology between the C. briggsae and C. elegans genomes as determined by WABA. Dark purple tracks indicate regions of strong homology (>70%). The sequence of the first conserved element of intron 10 is shown. The box indicates the part deleted and replaced with the sequence GAA in the del1.2 splicing reporter construct. The lower left part of the figure shows the results of ^32^P RT-PCR reactions with primers specific for the splicing reporter. Products for exon 9- or exon 10-containing messages are indicated. In embryos, only usage of exon 9 is detected for either reporter. At the L4 stage, 34% of the wild-type reporter messages contain exon 10 while del1.2 mutant messages contain only a trace amount of this exon.

There are many examples of regions of introns flanking alternatively spliced exons that function as *cis*-acting regulators of alternative splicing. We hypothesized that the reason that these high homology WABA-identified intronic elements have been conserved over 100 million years of evolution is that they may regulate alternative splicing. In order to test this hypothesis, we looked at the *C. elegans/C. briggsae* alignment of the alternatively spliced region of the C. elegans alpha(2) type IV collagen gene *let-2. Let-2* has two mutually exclusive alternative exons, exons 9 and 10, and their splicing pattern is evolutionarily conserved as far back as the distantly related parasitic nematode, Ascaris suum [35]. Messages incorporate either exon 9 or exon 10 in a developmentally regulated manner; embryos predominantly use exon 9, adults predominantly use exon 10, and there is a gradual shift in the usage of these two exons during the larval stages [36]. The WABA alignment of this 400-base intron identifies four conserved regions in the intron between exons 10 and 11 (Figure 2). To test the role of these conserved intronic elements in alternative splicing regulation, we employed a splicing reporter construct system containing the alternatively spliced region between exons 8 and 11 that mimics the developmental control of alternative splicing for this region when transformed into C. elegans [37]. We mutated the first conserved element in this intron and monitored the developmental regulation of this splicing. Deletion of this conserved element, in which 28 of 34 bases have been conserved between C. elegans and *C. briggsae,* results in a major reduction in the usage of exon 10 in L4 animals (unpublished data). The identical effect on splicing of an even smaller deletion within this element, del1.2, is shown in Figure 2. For the L4 time point, the developmental stage at which we should detect maximal exon 10 usage, only minimal splicing of this exon is detected. Since mutation of this element results in only minimal exon 10 inclusion, we hypothesize that either adults produce a splicing factor that interacts with this element to promote exon 10 splicing or that embryos produce a splicing repressor that inhibits exon 10 splicing. In the past, researchers have identified splicing regulatory elements by creating a series of mutations across an intron. In this computational approach, the interspecies genome alignments led us directly to a small element required for the developmentally regulated switch in this splicing.

### Analysis of a Database of Conserved Intronic Elements Flanking Alternatively Spliced Exons

As demonstrated in the previous section, evolutionarily conserved elements in introns flanking alternatively spliced exons can be important for alternative splicing. We used a computational approach to identify sequences that are more likely to occur in these conserved elements than in total intron sequences. As described above, we have identified 142 alternatively spliced genes in which the introns flanking an alternatively spliced cassette exon contain high homology regions with C. briggsae as defined by WABA. We extracted the highly conserved C. elegans sequences from these introns and put them into a database. The first or last seven nucleotides of introns were excluded from this dataset as these contain conserved signals for the constitutive splicing machinery [38]. This dataset contained 537 conserved elements of average length, 38.5 bases (minimum length, seven bases, longest, 231 bases, and median length, 28 bases) for a total of 20,675 bases. See Table S1 for a list of these elements and the alternatively spliced genes from which they were derived. We also generated a control dataset of all introns annotated in Wormbase genome sequence release WS 120.

Because many alternative splicing factors have binding preferences for relatively short sequence motifs [11,13,18,28–32], we decided to search the conserved intronic element dataset for short sequence motifs that appear more frequently here than in total introns. In order to identify sequences that are more prevalent in the conserved intronic regions bordering alternative exons, we counted the number of occurrences of every possible hexamer or pentamer motif in both our conserved intron element and total intron datasets. For each motif in the conserved element dataset, we determined the observed frequency, which was the count of each motif divided by the total number of possible motifs of that length in that dataset. We also determined an expected frequency based on the number of counts of each motif in the total intron dataset divided by the total number of possible motifs in that dataset. We then calculated an observed over expected (obs/exp) ratio for each motif, which was the observed frequency of each motif in the conserved element dataset divided by the expected frequency of each motif based on the total intron dataset. Table 1 shows the 40 top scoring pentamer and hexamer motifs in the conserved element dataset as determined by the obs/exp ratio value.

**Table 1:**
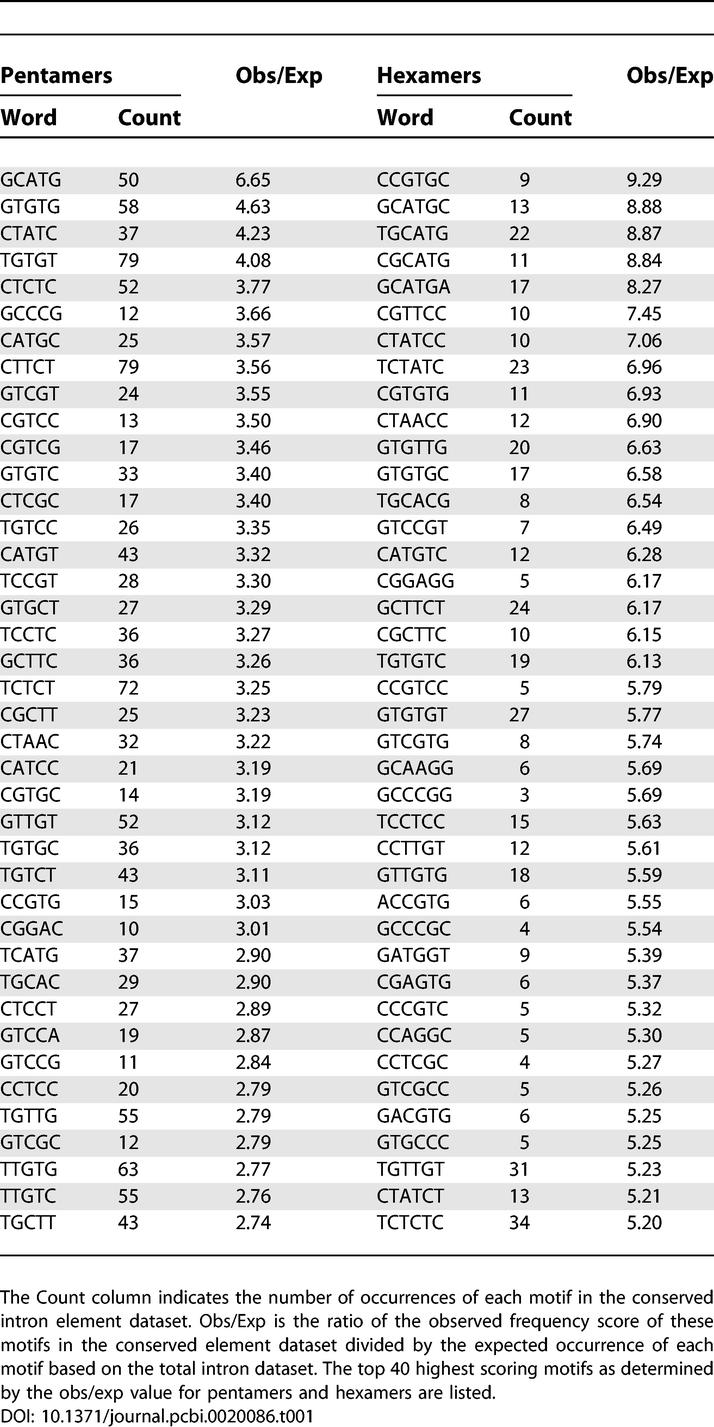
Pentamer and Hexamer Sequence Motifs That Occur More Frequently in Conserved Intronic Elements Flanking Alternatively Spliced Exons than in the Total Intron Dataset

Several previously identified mammalian splicing regulatory sequences are present in our lists of high-scoring hexamers and pentamers. This is encouraging because many of the known mammalian splicing factors have C. elegans homologs. For example, the sequence TGCATG and its subset pentamer GCATG have been shown to be important intronic regulators of splicing for the mammalian fibronectin and calcitionin/CGRP genes [14,15,39]. The mammalian FOX-1 protein selected the GCAUG sequence in a SELEX experiment, and it has been shown that the FOX-1 protein alters alternative splicing of several GCAUG-containing genes in transient transfection assays [13]. FOX-2, a homolog of FOX-1, has also been shown to regulate splicing of transcripts containing UGCAUG in neuronal cell culture [12]. FOX-1 was originally identified in C. elegans as a splicing factor of the transcript for the sex determination gene, *xol-1* [40]. Another well studied mammalian motif is that of intronic GU dinucleotide repeats, which have been shown to regulate splicing of the human *CFTR* gene [41] and serve as a binding site for the ETR-3 splicing regulatory factor [42]. CU dinucleotide repeats serve as a binding site for the PTB family members in the intronic regions that regulate inclusion of the N1 exon of *c-src* [43]. While targets for C. elegans alternative splicing factors might be inferred from their homology to mammalian proteins, very little experimental evidence exists for nematode splicing-factor binding sites. The C. elegans muscle-specific splicing factor SUP-12 regulates *unc-60* alternative splicing. This protein interacts with a GU-rich region, similar to the GU-rich motifs identified in Table 1, of an *unc-60* intron [44]. (For a review of other mammalian splicing factors with C. elegans homologs see [33].) While many of the high-scoring conserved motifs that we identified match known mammalian splicing regulatory sequences, we have also identified new motifs that may also be involved in pre-mRNA splicing regulation in mammals as well as nematodes.

One important aspect of C. elegans introns is that they are shorter than introns in vertebrates. Half of C. elegans introns are below 60 nucleotides in length, too short to be spliced in vertebrates [38]. However, many C. elegans introns are long and resemble vertebrate introns. In our alternatively spliced intron dataset, the median intron size is 263 bases (shortest is 43, longest is 10,719, and average is 561), indicating that these regulated introns generally belong in the larger intron class. We asked whether a similar analysis of the pentamers and hexamers found in the full introns flanking alternatively spliced exons would yield similar high-scoring motifs as our analysis of the conserved sequence subset of these introns. We did a pentamer and hexamer motif count of the full introns flanking the 147 alternatively spliced exons from which we previously extracted evolutionarily conserved elements. We counted the occurrence of every pentamer and hexamer in these full-length alternative introns. We ranked these motifs by the obs/exp ratio when we compared the occurrence of these motifs to their prevalence in our total intron dataset. The top 40 pentamer and hexamer motifs ranked by their obs/exp ratios are shown in Table 2. Many of the top scoring pentamers and hexamers that are overrepresented in conserved elements in introns flanking alternatively spliced exons are also overrepresented in total introns flanking alternatively spliced exons (compare Table 1 with Table 2). However, the obs/exp scores for the motifs in the alternative splicing introns is 2- to 3-fold lower than in the conserved element dataset extracted from these introns. For example, TGCATG has an observed to expected ratio of 3.35 in the whole introns that flank alternative exons but 8.87 for the conserved elements within those introns. TCTATC has a ratio of 2.55 in the total alternatively spliced intron analysis and 6.96 in the conserved element dataset analysis. The same holds true for high-scoring pentamers. CTATC goes from 1.85 for the observed to expected ratio in the introns flanking alternative exon dataset up to 4.23 in the conserved element dataset. From this analysis, the sequences of the introns flanking alternatively spliced exons can yield some data about potential pentamers and hexamers involved in alternative splicing regulation. However, limiting this analysis to WABA-conserved regions between C. elegans and C. briggsae can substantially improve the signal to help us identify splicing regulatory elements.

**Table 2:**
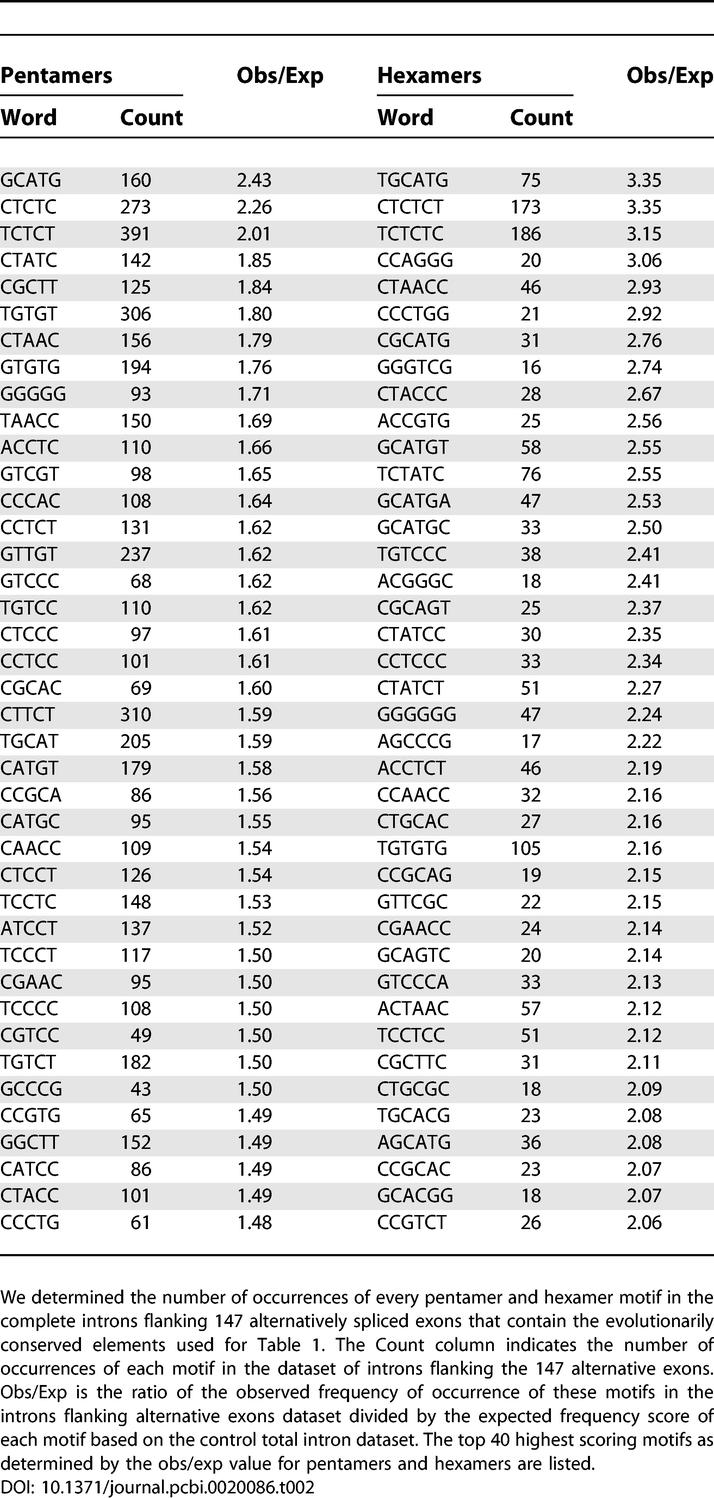
Pentamer and Hexamer Sequence Motifs That Occur More Frequently in the Introns Flanking Alternatively Spliced Exons than in Total Introns

We also used the database of introns flanking alternatively spliced exons to identify potential differences in the appearance of pentamer and hexamer motifs in the introns upstream or downstream of alternative exons. We grouped the introns upstream of the alternative exons and the introns downstream of the alternative exons separately into datasets and did motif counts and determined the obs/exp ratio for each when compared to the total intron dataset. These results are shown in Tables 3 and 4, with the top scoring pentamers and hexamers in the introns upstream and downstream of alternative exons ranked by their obs/exp ratios. Some of the high- scoring motifs show very little preference for the upstream or downstream intron. For example, high-scoring hexamer TGCATG has an obs/exp ratio of 3.90 in the downstream intron and 3.06 in the upstream intron. Others show a strong preference. CTCTCT and TCTCTC, likely binding sites for PTB, have obs/exp ratios of 5.15 and 4.84, respectively, in upstream introns, but 1.36 and 1.30 in downstream introns. TCTATC, which will be analyzed below, also has a preference for upstream introns, with an obs/exp ratio of 3.25 in upstream introns and 1.79 in downstream introns. Conversely, CCAACC has a strong preference for downstream introns, with an obs/exp ratio of 3.17 in the downstream intron and 1.29 in the upstream intron. In addition to identifying potential binding sites for splicing regulatory factors, these differences in appearance in introns upstream or downstream of alternatively spliced exons may hold some keys to function.

**Table 3:**
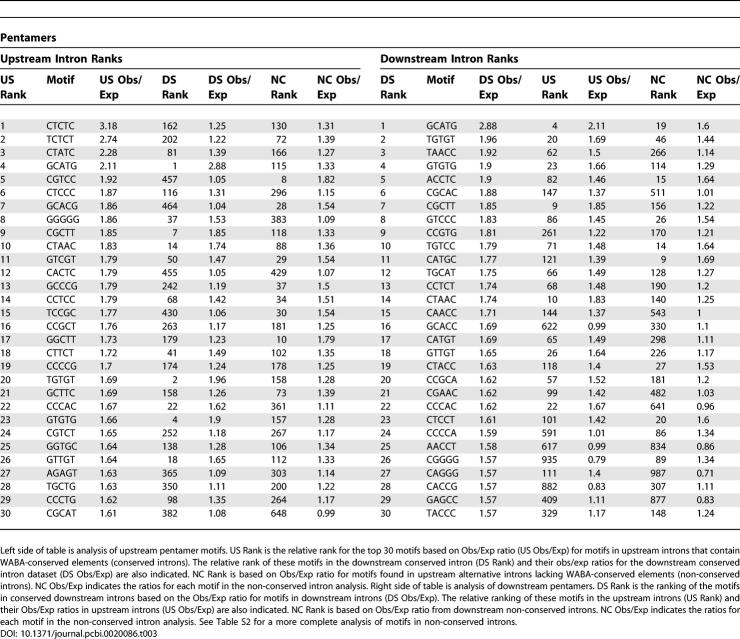
Comparison of the Occurrence of Pentamer Motifs in Introns Upstream versus Downstream of Alternative Cassette Exons

**Table 4:**
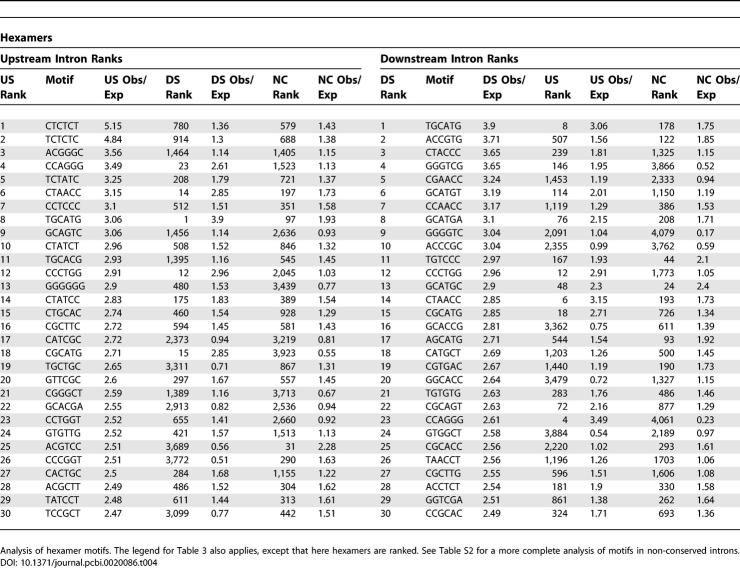
Comparison of the Occurrence of Hexamer Motifs in Introns Upstream versus Downstream of Alternative Cassette Exons

To determine whether these pentamers and hexamers are found in introns flanking all alternative cassette exons or are specific for those introns with WABA-conserved elements, another dataset was constructed from the introns flanking the alternative cassette exons of 307 genes lacking WABA-defined evolutionarily conserved intronic elements. A motif search similar to that described in the previous paragraph was conducted by dividing the new, non-conserved intron dataset into two sets: introns upstream of alternative exons and those downstream. Motifs were counted for each of the sets and compared to the total intron dataset to generate an obs/exp ratio and corresponding ranking for each element. It can be seen from Table 4 that the obs/exp scores of all of the top 30 hexamer motifs identified in conserved introns drop in the analysis of non-conserved introns, suggesting the motifs are indeed specific to introns containing conserved regions. For example, our top ranking hexamer from the conserved intron analysis, TGCATG, has an obs/exp score of 3.9 in downstream introns and 3.06 upstream. This same motif only scores 1.75 in downstream and 1.93 in upstream in non-conserved alternative introns (Table 4). For CTCTCT and TCTCTC, our two top hexamer upstream motifs in introns with WABA-conserved elements, the obs/exp ratio drops over 3-fold in this analysis, and they rank as 579 and 688, respectively, in the upstream non-conserved intron set (Table 4). Conversely, top scoring motifs from the non-conserved intron database are not highly represented in the conserved intron dataset. For example, GGCCAC, with an obs/exp ratio of 4.36 (Table S2) was the top scoring motif in the upstream non-conserved dataset. Strikingly, this same motif has an obs/exp score of 0.11 in total introns that contain WABA-conserved elements upstream of an alternatively spliced exon (unpublished data). This dramatically different representation of top scoring pentamers and hexamers in introns containing WABA-conserved elements versus those that do not suggests that the presence or absence of intronic WABA-defined conservation may define two distinct classes of alternatively spliced exons with distinct splicing regulatory mechanisms.

To ensure that the motifs identified in the conserved element dataset were truly overrepresented in this region and not a consequence of WABA creating bias towards GC-rich regions in the AT-rich nematode genomes, we analyzed the base composition of our datasets. The GC content of our WABA-defined conserved element dataset was 39%, the total intron dataset that contained these conserved elements was 34%, and the total intron reference dataset was 32%. While this slight variation in GC content might suggest that the WABA HMM may have created a bias, two arguments can be made against this. The first is that the high-scoring motifs in the WABA-conserved region were also highly represented in the total introns containing these motifs (Table 2), consistent with these results not being derived from bias in GC content. The second is that GC enrichment bias is not observed in many of our highest scoring motifs. For example, TCTATC has a GC content of 33%, yet is highly enriched in conserved elements.

### The Candidate Motifs (T)GCATG and (T)CTATC Are Required for *unc-52* Alternative Splicing Regulation

**Figure 3:**
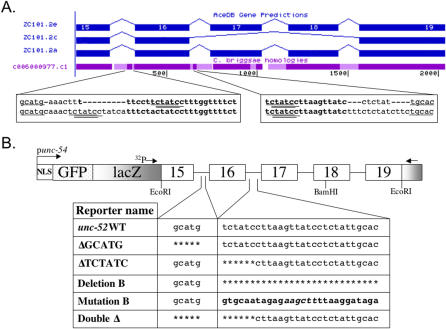
Conserved Intronic Elements of *unc-52* Contain Putative Regulators of Alternative Splicing (A) C. elegans and C. briggsae sequence alignment is shown for the alternatively spliced portion of *unc-52*. Not all spliced isoforms are predicted by Wormbase software (blue); see Figure 4A for observed alternative splicing patterns*.* PhastCons sequence alignment is shown with WABA-designated conservation in bold. Upper line of sequence is *C. elegans; C. briggsae* is below. High-scoring conserved motifs identified in our pentamer/hexamer analysis of conserved intronic elements flanking alternatively spliced exons, GCATG, TCTATC, CTATCC, CTATC, and TGCAC are underlined. (B) Diagram of alternative splicing reporter constructs for testing putative *cis*-regulatory splicing motifs. Part of exon 15 through part of exon 19 of *unc-52* was cloned into a GFP/lacZ fusion vector with an *unc-54* promoter and nuclear localization sequence suitable for expression in *C. elegans.* Site-directed mutagenesis of the wild-type substrate was performed in order to test putative *cis*-splicing regulatory elements*.* A table of the splicing reporter constructs and their alterations is shown. Asterisks denote highly conserved intronic nucleotides deleted by site-directed mutagenesis. To maintain the intron length, yet remove motifs in question, a reporter was also made in which native sequence was replaced with the reverse complement sequence (shown in bold) and a HindIII site (italics) for diagnostic purposes.

The *C. elegans unc-52* gene is a homolog of the mammalian extracellular matrix protein perlecan and contains 37 exons. Exons 16, 17, and 18 are alternatively spliced in a complex and regulated manner. These three exons each encode an Ig protein motif and can be included or skipped in any number of combinations in the final *unc-52* mRNA transcript [45]. RT-PCR analysis shows *unc-52* is also alternatively spliced in *C. briggsae,* suggesting the splicing pattern may be controlled by similar methods (unpublished data). The use of a subset of these alternatively spliced forms is controlled by the splicing regulatory protein, MEC-8 [46,47]. The genetic regulation of *unc-52* alternative splicing makes this gene an attractive model for studying splicing regulation. Most importantly for this analysis, the introns flanking either side of alternative exon 16 contain regions of high nucleotide conservation as identified by WABA. A number of the top scoring pentamers and hexamers from our conserved element motif analysis are found in these conserved regions (Figure 3A).

**Figure 4:**
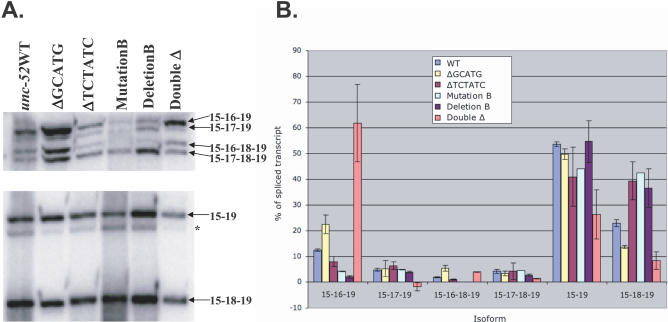
Deletion or Mutation of Top Scoring Pentamers or Hexamers Alters *unc-52* Alternative Splicing (A) Autoradiogram of *unc-52* reporter gene alternative splicing. Left side shows ^32^P RT-PCR analysis (with BamHI digest) performed on strains carrying each of the reporter constructs described in Figure 3B. The reporter is indicated at top of gel. Table on right shows the relative percentage of each alternatively spliced isoform produced from the indicated in vivo splicing reporter constructs. Arrows point to the corresponding band on the gel. Asterisk denotes a non-specific band resulting from RT-PCR. (B) Graphical summary of *unc-52* in vivo splicing reporter assays. Horizontal axis indicates spliced mRNA isoform. Vertical axis represents the relative percentage of each isoform. Color key on figure indicates which bars correspond to which strain. Standard deviation and mean values were calculated based on a minimum of three independent RNA extractions and subsequent RT-PCRs for each reporter.

To test the role of these motifs in alternative splicing of *unc-52,* we created a muscle-expressed alternative splicing reporter transgene derived from this region (Figure 3B). Expression studies using the native *unc-52* promoter to drive GFP expression have indicated that this extracellular matrix protein is expressed in the muscle and hypodermis [47,48]. However, studies of the *unc-52* splicing regulator *mec-8* have suggested that *mec-8* function on *unc-52* alternative splicing is mostly focused in hypodermal tissues [47]. Our intention is to mimic the native gene's splicing as closely as possible, so we tested whether the alternative splicing of our muscle-specific *unc-52* reporter could be regulated by *mec-8*. RT-PCR analysis of our wild-type *unc-52* alternative splicing reporter in *mec-8(+)* (wild-type) and *mec-8(e398)* (mutant) backgrounds indicates a dramatic difference in splicing (Table S3). When the reporter is spliced in a *mec-8(e398)* mutant that lacks its RNA-binding domain, an increase in exon 18-containing transcripts is observed, as well as a 75% drop in abundance of 15-16-19 and 15–19 isoforms. This closely mimics the effects of *mec-8(e398)* on native *unc-52* splicing [46]. These results demonstrate that the tissue-specific expression of our *unc-52* reporter is an adequate representation of the alternative splicing of the native gene, and that it is also under the control of the splicing factor MEC-8.

In order to test the role of evolutionarily conserved intronic elements and motifs in the regulation of *unc-52* alternative splicing, we created *unc-52* alternative splicing reporter constructs with different combinations of deletions in these sequences and monitored their in vivo splicing by RT-PCR. Due to the similar size of exons 17 and 18, a restriction digest with BamHI was performed on the RT-PCR products. Spliced isoforms containing exon 18 would be cleaved by BamHI and thus run at smaller sizes on the gel (Figures 3B and 4A). A summary of these results is shown in Figure 4. All quantitation is based on the analysis of a minimum of three different RNA isolations from the indicated strains. In general, splicing reporters with deletions of the conserved intronic sequences flanking either side of exon 16 seemed to have the largest effect on three specific isoforms of mature *unc-52* mRNA, those containing exons 15-16-19, 15-16-18–19, and 15-18-19 (Figure 4B).

Our pentamer and hexamer motif analysis of conserved intronic elements flanking alternatively spliced exons identified GCATG and TGCATG as high-scoring motifs likely to be found in introns flanking either side of an alternative exon. Deletion of the GCATG upstream of alternative exon 16 from the *unc-52* splicing reporter resulted in nearly a doubling of the proportion of reporter transcripts containing exon 16 and a drop in the proportion using 15-18-19. This suggests that GCATG may play a role in repressing the inclusion of exon 16 (Figure 4B).

A conserved intronic element just downstream of exon 16 contains another of our top scoring motifs, (T)CTATC. A deletion of this TCTATC had the opposite effect of deleting the upstream intronic GCATG; the proportion of exon 16-containing transcripts decreased while the amount of 15-18-19 increased. A larger deletion of this downstream motif (Deletion B) or a replacement of the motif with the reverse complement sequence (Mutation B) amplified this effect as summarized in Figure 4B. Specifically, an approximately 75% reduction in exon 16- containing transcripts relative to wild-type can be seen when this conserved region is altered in the Mutation B construct. A similar drop of almost 85% is seen when this conserved element is deleted in the Deletion B reporter construct. This indicates that TCTATC is part of a regulatory element that enhances the inclusion of exon 16, and its loss by mutation leads to a decrease in exon 16 inclusion.

Perhaps most interesting was the effect produced by the double deletion of two conserved motifs flanking exon 16: the upstream GCATG and the downstream TCTATC. Individual deletions of these two motifs suggested they work in opposition to include or exclude exon 16. However, the double mutant causes a shift in splice pattern similar to, but much more dramatic, than that seen in the ΔGCATG construct (Figure 4B). The double mutant had such a dramatic increase in 15-16-19 levels that it became the new predominant isoform, making up 61% of the mRNAs. This result suggests that the conserved motifs on either side of exon 16 are responsible for the inclusion of this exon and that they work in collaboration and as part of a larger regulatory network to produce a multilayered regulation of *unc-52* splicing. In general, changes to the evolutionarily conserved intronic elements flanking exon 16 did not alter the relative amounts of the 15-17-19, 15-17-18–19, and 15–19 isoforms dramatically. These elements flanking exon 16, for the most part, appear to regulate the decision to form either the 15-16-19 isoform or the 15-18-19 isoform (Figure 4B).

## Discussion

Our comparative genomic analysis of C. elegans and C. briggsae allowed us to identify 142 alternatively spliced genes that exhibit regions of high nucleotide conservation in introns flanking alternatively spliced exons. These conserved regions were then analyzed for pentamer and hexamer motifs present at a statistically higher level than found in total introns. Many of the high scorers on these lists matched known mammalian splicing regulatory elements. This indicates that this approach can find alternative splicing regulatory sequences, and it is consistent with our observations that there are C. elegans homologs for the major mammalian splicing regulatory factors (unpublished data). In addition to finding known splicing regulatory sequences, this approach identified potentially novel splicing regulatory sequences. We have confirmed that the sequence (T)CTATC is important for alternative splicing regulation of the *unc-52* gene.

The limited sequence conservation between C. elegans and C. briggsae in introns flanking alternatively spliced exons contrasts with that seen in mammalian interspecies genome alignments. In mouse/human alignments of alternatively spliced human cassette exons, it was found that the 100 nucleotides of intron sequence flanking either side of alternative cassette exons tend to be highly conserved [21,22]. It is unclear from these mouse/human analyses which specific portion(s) of the introns immediately flanking these alternatively spliced exons are essential for alternative splicing regulation. Our analysis indicates that the WABA-identified regions of intronic homology flanking alternatively spliced exons are likely to be important for alternative splicing regulation. Therefore, the *C. elegans/C. briggsae* evolutionary distance, along with the sensitivity of WABA, provides for a more simplified method for pinpointing splicing regulatory sequences. One of the interesting questions resulting from our identification of intronic alternative splicing regulatory motifs in C. elegans is whether these will also function to regulate alternative splicing in mammals. It has been suggested that there is a difference in frequency of usage of constitutive intronic splicing enhancers between mammals and fish, which can explain why fish introns are not always spliced efficiently in mammalian cells [49]. No similar study for alternative splicing regulatory elements has yet been done. Our data on C. elegans alternative splicing regulatory elements suggest that there are many similarities in alternative splicing regulation across metazoans. This is seen in the fact that many of the motifs we identified in worms are known to regulate alternative splicing in mammals. Whether novel elements we have identified, such as TCTATC, can also function in mammals has yet to be determined.

The identification of (T)GCATG as a top scoring alternative splicing regulatory motif demonstrated the usefulness of this approach, as many examples of this motif as a splicing regulatory element are present in the literature [14,15,50]. (T)GCATG is statistically overrepresented in introns downstream of alternative exons conserved between human and mouse genomes [21,22] and may have a spatially conserved role in directing splice-site choice [23]. On the other hand, in vitro studies have demonstrated that the splicing factor FOX-1 affects splicing of transcripts with GCATG either upstream or downstream of an alternative exon [13]. One interesting finding of our C. elegans work is that GCATG in introns flanking alternatively spliced exons shows no preference for being upstream or downstream of alternative exons. This may be evidence that while the motifs that regulate alternative splicing between mammals and nematodes may be the same, they may be used in different ways to promote splicing.

A recent analysis of the C. elegans genome used a support vector machine to identify predictive features of C. elegans alternative exons [51]. One of the predictive features of an alternatively spliced exon that they identified is the presence of several different hexamers in the introns surrounding alternative exons. In their supplemental materials (http://www2.fml.tuebingen.mpg.de/raetsch/projects/RASE) they list the top 12 predictive hexamers in the introns upstream and downstream of the alternative exon. Comparison of their high-scoring results with our conserved element analysis indicates that many of the top scorers in both approaches are identical (for example, TGCATG, CTAACC, GTGTGT, CTCTCT, and TCTATC). That both their support vector machine and our comparative genomics methods yielded similar results helps to confirm the validity of both methods.

Comparing the motif searches from different classes of intronic sequence (total introns, WABA-conserved elements, entire conserved introns flanking alternative exons, and non-conserved introns flanking alternative exons) brought about a number of insights concerning the presence of certain motifs in introns. First, it can be noted that many of these sequences are still found, although at lower frequency, in total introns as well as those that are alternatively spliced. They may therefore, in a method analogous to SR protein-binding sites in exons, play a role in constitutive as well as alternative splicing (SR proteins reviewed in [52]). Second, the comparison of motif analyses of introns lacking conserved elements and those containing WABA conservation reveals two dramatically different lists of overrepresented motifs. This suggests that there may in fact, be two separate classes of cassette exon splicing regulation, and WABA can help us distinguish between them.

Our splicing reporter system allowed us to directly test the role of the conserved elements GCATG and TCTATC flanking alternative exon 16 of the *unc-52* gene in vivo. Deletion of either of these elements individually led to measurable but opposing effects on the inclusion of this exon (Figure 4). These first results suggested the two *cis* elements might be counter-balancing each other to regulate splicing in this region. However, the creation of a double deletion of these two elements demonstrated that this was a naive assumption. The splicing program exhibited by the double deletion construct was similar to that seen from the ΔGCATG construct: an upregulation of the exon 16-containing transcripts. However, the proportional increase of these isoforms was far more dramatic in the double than the single deletion. The 15-16-19 isoform became the predominant spliced transcript, as opposed to 15–19 seen for all other splicing reporter constructs. This striking result is a good reminder that although these motifs can work independently and their deletion may produce measurable effects on splicing, they may work in a combinatorial way in vivo, and the effects of multiple deletions of important elements may be hard to predict. This type of combinatorial multifactor splicing regulatory mechanism has been described for several vertebrate genes including *c-src* [11] and cardiac troponin T [17]. While our comparative genomics approach gives us a method of identifying putative *cis*-regulatory elements of alternative splicing and our in vivo reporter assay provides a method for directly testing candidates, we still need more experimental information before we can predict the combinatorial effects of these elements on splicing.

Our comparative genomics analysis has not only provided a method for identifying intronic *cis-*regulatory elements, but also a starting point to investigate the mechanisms by which they control splicing. Consistent with current models of alternative splicing, *cis* elements are predicted to be binding sites for *trans* factors. Our identification of the novel splicing regulatory sequence (T)CTATC will lead us to subsequent experiments to identify its potential protein partner. The lists we generated of potential splicing regulatory elements include many sequences that have yet to be tested for a role in alternative splicing regulation. Experiments still need to be performed, similar to those done on *unc-52* and *let-2,* in which these elements are tested for a functional role in alternative splicing regulation. Due to the rapid release of new alignment programs and genomes, our list of conserved pentamers and hexamers will likely see some adjustments before all potential regulatory motifs have been tested. WABA has a limitation of needing to see relatively large sequence alignments in order to call a region as high homology (the minimum WABA high homology sequence run we detected was seven consecutive identical nucleotides). WABA high homology regions in introns flanking alternatively spliced exons were only detected for approximately 25% of alternative cassette exons in our dataset. The newer alignment algorithm PhastCons [53], which uses an HMM to align multiple genomes using a smaller window size than that of WABA, can allow us to more accurately pinpoint smaller regions of conserved nucleotides within introns flanking additional alternatively spliced exons (unpublished data). The release of additional nematode genomes such as Caenorhabditis remanei should provide us with the prospect of creating a three-way nematode genomic alignment, allowing us even more accuracy in our regulatory motif predictions. By systematically testing the pentamers and hexamers on our list of conserved motifs, we may be able to confirm more alternative splicing regulatory motifs. A compilation of these results will provide us with a better comprehension of the rules governing this complex and essential process.

## Materials and Methods

### Database construction.

A control dataset of introns was obtained by downloading from the UCSC Genome Browser (http://www.genome.ucsc.edu) all of the introns in the C. elegans genome as annotated in Wormbase release WS120. From the sequences of these 118,492 introns, we removed the first and last seven nucleotides to avoid constitutive splicing signals. We then counted the number of occurrences of every possible hexamer or pentamer motif in the database by using the EMBOSS Compseq program [54]. For each motif in the database, we assigned a frequency score that was the number of occurrences of each motif divided by the total number of possible occurrences of motifs of that length in the dataset. This score for each possible motif in the control set was used in our analysis of both the introns that flank alternatively spliced exons and the conserved elements in these introns as our expected score. In order to obtain the obs/exp ratio for each motif in a test dataset, we divided the observed frequency score for each motif in that dataset by the expected frequency score for each motif based on the total intron control dataset.

### Analysis of alternative exons.

C. elegans and C. briggsae alignments for the 147 alternative spliced exons with intronic conservation were obtained from the UCSC Genome Table Browser conservation track [55]. The mean nucleotide percentage identity was computed from this alignment along with the number of insertions and deletions. In order to obtain the mean amino acid identity, all the 147 exons were compared to the complete C. briggsae genome (WormBase cb25.agp8) using TBLASTX [56], and the mean amino acid identity was then calculated from these alignments.

### Generation of *let-2* splicing reporter constructs.

We have previously described the development of an in vivo splicing reporter for the alternatively spliced region of the *let-2* gene fused to GFP and expressed in muscle cells [37]. Site-directed mutagenesis of the wild-type reporter for *let-2* alternative splicing from that paper was used to delete the core of the first conserved element, indicated in Figure 2, and replace it with GAA. Methods for detecting alternative splicing of this transcript with ^32^P-labeled primers using RT-PCR, denaturing polyacrylamide gel electrophoresis, and autoradiography have been previously described [37].

### Generation of *unc-52* splicing reporter constructs.

The exon 15–19 region of *unc-52* was PCR-amplified from wild-type (N2) C. elegans genomic DNA using primers 5′-GGAATTCGATGAGTACATCTGTATCGC and 5′-GGAATTCACATCTGAACTGATGTCGCTC for cloning into the vector pPD96.02, developed by Andrew Fire's lab. This vector contains the *unc-54* body wall muscle-specific promoter driving expression of a green fluorescent protein (GFP)/β galactosidase (lacZ) fusion protein with an N-terminal nuclear localization signal derived from SV40 T antigen. The final 127 nucleotides of exon 15 through the first 173 nucleotides of exon 19 of *unc-52* and all the genomic sequence in between were cloned into a unique EcoRI site, 16 codons before the end of the lacZ open reading frame in the final exon of the fusion protein. The Kunkel method of site-directed mutagenesis was used to create the larger deletion and mutations for the plasmid constructs Mutation B and Deletion B [57]. Pentamer and hexamer deletion constructs of the conserved *unc-52* intron sequence were created using the Quickchange site-directed mutagenesis kit (Stratagene, La Jolla, California, United States) according to the manufacturer's instructions. Animals carrying these constructs as extra-chromosomal arrays were generated by standard injection/transformation [58]. Transformed N2 animals were identified by GFP-expression in nuclei of body wall muscle cells.

### Growth of C. elegans strains and isolation of RNA.

C. elegans strains were grown on NGM agar plates using standard methods [59]. RNA was extracted from worms as previously described [60]. Strain CB938 carrying the *mec-8(e398)* mutant allele was obtained from the C. elegans Genetics Center, a National Institutes of Health funded Center for Research Resources at the University of Minnesota.

### Production of cDNAs.

cDNAs of the *unc-52* splicing reporters were made in 25-μl reaction mixtures. The annealing step contained 3 μg total RNA and 25-pmol oligodeoxynucleotide primer complementary to the lacZ construct (5′- GTTGAAGAGTAATTGGACTTA-3′ for C. elegans and 5′- AACTGGTGTCGCTCTCCT-3′ for C. briggsae) in 17-μl final volume. These were heated to 94 °C for 2 min and cooled to room temperature for 10 min. The rest of the reverse transcription reaction components were added to reach a final volume of 25 μl. The final reaction mixtures consisted of 1 mM each of dATP, dCTP, dGTP, and dTTP; 1 U RNA Guard (Promega, Madison, Wisconsin, United States); 1 × AMV RT buffer (Promega); and 10 U AMV reverse transcriptase (Promega). Reactions were incubated at 37 °C for 1.5 h and stored at −20 °C.

### Polymerase chain reaction.

1.0 μl of the cDNA reaction mixture was used as the template in 25-μl PCR reaction mixtures. 1.0 pmoles of the same oligonucleotide used in the reverse transcription reactions was added to PCR reaction mixtures along with ^32^P-labeled 5′ lacZ vector-specific primer (5′-CTGGAGCCCGTCAGTATCGGC-3′ for C. elegans and 5′-ATTCGTTGCTGGGTCCCAGG-3′ for C. briggsae). The reaction mixtures also contained 1 × PCR buffer, 0.25 mM of each of the four dNTPs, and *Taq* DNA polymerase. The 5′ oligo was labeled with (γ-^32^P) ATP by T4 polynucleotide kinase. Reaction mixtures were incubated for 25 cycles at 94 °C for 1.0 min, 59 °C for 1.0 min, and 72 °C for 1.0 min. 2.0 μl of PCR product were digested with the restriction enzyme BamHI. There is a unique BamH1 site in *unc-52* exon 18, and this digestion step allows us to distinguish the different alternatively spliced isoforms more clearly on the gel. Digested PCR products were separated on 40-cm long, 0.4-mm thick, 5% or 6% polyacrylamide urea gels in TBE buffer. Gels were dried onto filter paper. These were then visualized using a Molecular Dynamics PhosphorImager (Sunnyvale, California, United States). Relative splice-site usage was quantified using ImageQuant software as previously described [37].

## Supporting Information

Table S1 Complete Database of Highly Conserved Intronic Elements Used in Motif Analysis Column (A) indicates C. elegans gene name. Columns (B–AI) list the WABA high homology sequences found in C. elegans introns flanking alternative exons of the corresponding gene.
(51 KB XLS)

Table S2 Complete Table Detailing Ranks of Pentamer and Hexamer Motifs in Introns Lacking Evolutionary Conservation Surrounding 307 Alternatively Spliced Exons On both worksheets: Column (A) represents rank of motif from search based on obs/exp frequency in introns lacking evolutionary conservation downstream of alternative introns. Column (B) denotes motif and (C) contains the number of times the motif was observed in the dataset. (D) The observed frequency of the motif in the dataset. (E) Contains the expected frequency of motif based on total intron dataset. Column (F) is the ratio of observed frequency to expected frequency. Columns (H–M) are data from the same analysis in the same order for upstream introns.
(1.2 MB XLS)

Table S3 Each Row in the Table Represents a Different Spliced Isoform of the *unc-52* Reporter Constructs, and Each Column Denotes the Reporter Being Monitored The first six columns show the relative percentage of alternative splice-site usage of the specified reporter in a *mec-8(+)* background as quantified from ^32^P RT-PCR*.* The seventh column shows results of the wild-type sequence reporter that has been crossed into *mec-8(e398)* mutant animals.
(185 KB TIF)

## Abbreviations

EST: expressed sequence tag
HMM: hidden Markov model
obs/exp: observed over expected
pre-mRNA: precursor messenger RNA
PTB: polypyrimidine tract binding protein
WABA: Wobble Aware Bulk Aligner
